# Incomplete intestinal obstruction as an initial and persistent presentation in chronic inflammatory demyelinating polyneuropathy: A case report

**DOI:** 10.1097/MD.0000000000013538

**Published:** 2018-12-10

**Authors:** Long Wang, Xiao-Zheng Yuan, Xue-Min Zhao, Fu-Yu Wang, Yu Wang

**Affiliations:** a Department of Neurology, The First Affiliated Hospital of Anhui Medical University, Hefei; b Department of Neurology; c Department of Pharmacy, General Hospital of Wanbei Coal and Electrical Group, Suzhou, China.

**Keywords:** autonomic symptoms, chronic inflammatory demyelinating polyneuropathy, glucocorticoid, intestinal obstruction, intravenous immunoglobulin

## Abstract

**Rationale:**

Autonomic symptoms are not uncommon in chronic inflammatory demyelinating polyneuropathy (CIDP), but they are mostly mild and transient and are overshadowed by somatic manifestations. Here, we report a very unusual case of CIDP with severe autonomic symptom, intestinal obstruction, as initial and persistent symptom which responded well to high-dose glucocorticoid and intravenous immunoglobulin treatment.

**Patient concerns:**

We described a patient with CIDP with precedent and long-lasting incomplete intestinal obstruction. Clinical manifestations were precedent and chronic abdominal pain, distension and constipation, and later numbness and weakness of lower and upper limbs. Radiograph showed incomplete intestinal obstruction, cerebrospinal fluid (CSF) showed albuminocytological dissociation and electromyography indicated neurogenic lesion.

**Diagnoses:**

CIDP with incomplete intestinal obstruction was diagnosed based on the history, related symptoms, typical abdominal radiograph, CSF albuminocytological dissociation, and electromyographic findings.

**Interventions:**

The patient was treated with intravenous methylprednisolone and immunoglobulin.

**Outcomes:**

After treatment, the intestinal obstruction disappeared and the somato-symptoms improved greatly and gradually.

**Lessons:**

This case highlights the need for diagnostic vigilance in cases of incomplete intestinal obstruction of unknown cause. We recommend CSF and electromyography examination in view of rare but possibility of CIDP.

## 1 Introduction

Chronic inflammatory demyelinating polyneuropathy (CIDP) is the most common treatable neuropathy, with a prevalence ranging from 1 to 9 cases per 100,000. CIDP typically presents as either relapsing-remitting or progressive neuropathy with motor weakness and sensory disturbance affecting proximal and distal limbs and its course usually develops over 2 months.^[1]^ On the other hand, autonomic symptoms presenting as cardiovagal, sudomotor, vasomotor, genitourinary, and gastrointestinal dysfunction are not uncommon in CIDP, they may precede or accompany the somatic manifestations but are mostly mild and transient and are overshadowed by the somatic manifestations.^[2]^ In very rare cases, such as in cases with pandysautonomia, the autonomic symptoms due to demyelinating polyneuropathy may be severe. In these pandysautonomia cases, gastrointestinal dysfunctions are common and present as vomiting, constipation, and alternative diarrhea and constipation, of which some may meet the diagnosing criteria for intestinal obstruction.^[3]^ But in all reported pandysautonomia cases, severe gastrointestinal dysfunctions were not isolated from other autonomic symptoms and were acute onset (acute inflammatory demyelinating polyneuropathy) mainly in childhood or adolescence.^[3–5]^ Concerning the gastrointestinal manifestation in CIDP, it has never been reported that an intestinal obstruction presented as an initial symptom. Here, we described a 55-year-old patient who developed an incomplete intestinal obstruction preceding the somatic manifestations of CIDP and the gastrointestinal as well as the motor and sensory dysfunction persisted for long time until glucocorticoid treatment.

## 2 Case presentations

A 55-year-old female civil servant was admitted to the neurology department of our hospital because of hypogastralgia and hard defecation for 4 months and progressive numbness and weakness of 4 limbs for 3 months. At the beginning of abdominal pain and hard defecation, she was admitted to gastroenterology department of other hospital. Blood routine test, liver and kidney function, electrolyte, amylase, lipase, and other examinations were conducted and showed no abnormal findings. Abdominal plain radiograph showed multiple small bowel air-fluid levels suggesting an incomplete intestinal obstruction. The patient accepted gastrointestinal decompression and rehydration treatment for 1 week and the abdominal pain improved. However, constipation did not recover. Further colonoscopy examination revealed no abnormality. At the this time, that is, 2 weeks after the beginning of abdominal pain and hard defecation, the patient presented numbness below the left ankle which attracted no attention from the doctors and she was discharged. Ten days after discharge, the left lower limb numbness ascended to the level of the knee joint and the right lower limb toes became numb. And it was becoming a little difficult to get up and down stairs at this time due to the weakness of the lower limbs. At a local clinic, she was prescribed with vitamin B1 and mecobalamin tablets but the numbness and weakness of the lower limbs were still deteriorating. Two weeks later, that is, about one-and-half month after the beginning of abdominal pain and hard defecation, the patient developed numbness at the extremities of both upper limbs but the upper limbs were basically normal in holding objects and doing housework. Nevertheless, the muscle strength of both upper and lower limbs was still declining gradually. When the patient came to our department, she had difficulty in walking and needed wheelchair and it was difficult for the upper limbs to hold bowl and chopsticks steadily. And she gradually developed a band feeling on the chest and abdomen but she had no difficulty in breathing and urination. During the disease course, no swallowing problem was reported but the abdominal pain and distension and constipation were persistent. Therefore, it was necessary to assist the defecation with glycerol enema and glycerinum.

The patient denied diarrhea or respiratory infection before this event. The patient did not take any other medicine except for nifedipine (20 mg twice a day) to control hypertension for 5 years. She denied any history of previous abdominal surgery. Family history was not significant. The patient did not have any other history of medical treatment except for the hypertension and its treatment.

On general examination, the patient was ill looking and mildly dehydrated. Her body temperature was 36.8°C but her extremities were cold. Blood pressure was 126/70 mm Hg, Pulse rate 80 per minute. Abdominal examination revealed gross distention with sluggish bowel sounds. No abnormality was detected in cardiovascular, and respiratory system and no congenital anomalies found. On neurological examination, cranial nerves were normal. Muscle strength testing showed bilateral upper limb distal muscle strength grade 3 and proximal muscle strength grade 4, left lower limb muscle strength grade 3, and right lower limb muscle strength grade 3 plus. Bilateral Achilles tendon reflex and knee reflex disappeared, bilateral biceps and triceps reflex weakened, and bilateral pathological signs were negative. Bilateral limb muscle tension was decreased. Bilateral gastrocnemius tenderness was detected. Bilateral sensation was impaired below elbows and knees. Nobody plane under which sensory disturbance was detected.

**Figure 1:**
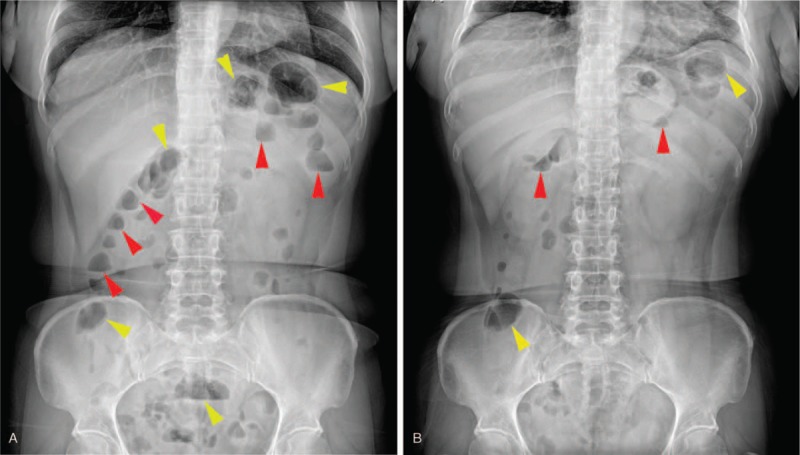
Abdominal plain radiography revealed amounts of air-fluid levels (red arrowheads) and massive gas accumulation in the dilated intestinal canal (yellow arrowheads) before glucocorticoid treatment (A), and fewer air-fluid levels (red arrowheads) and massive gas accumulation in the dilated intestinal canal (yellow arrowheads) after glucocorticoid treatment (B).

Routine blood tests as well as liver, kidney, and thyroid function were normal, and blood electrolyte, glucose, lipid, rheumatoid factor, tumor makers, and immune disease-associated autoantibodies all in normal ranges. For cerebrospinal fluid (CSF) examination, the fluid was colorless and transparent with the pressure of 130 mm H_2_O. The number of nucleated cells was 16 × 10^6^/L (normal range, 0–8 × 10^6^/L), glucose and chlorides normal, protein 2541 mg/L (normal range, 150–450 mg/L), immunoglobulins (Ig) G 546 mg/L (normal range, 10–40 mg/L), IgA 104 mg/L (normal range, 0–6 mg/L), and IgM 29.5 mg/L (normal range, 0–13 mg/L). Abdominal plain radiograph in standing posture showed multiple air-fluid levels and a “whirlpool” image into the right iliac fossa suggesting an incomplete small intestinal obstruction (Fig. 1A). For electrophysiological examination, the motor nerve conduction velocity (MNCV) of bilateral median, ulnar and tibia nerves as well as the left musculocutaneous nerve were decreased, and the latency prolonged. F wave of bilateral ulnar nerve and H reflex of bilateral tibial nerve could not be evoked. As the patient had a band feeling on the chest, cervical and thoracic magnetic resonance imaging (MRI) examinations were conducted and revealed normal.

The patient was considered to be affected with CIDP. Methylprednisolone was given at 500 mg a day for 5 days and reduced to 250 mg a day for 3 days. At this time, the patient's abdominal pain and distension disappeared and the constipation improved, but the motor and sensory dysfunction improvement was not significant. Re-examination of the abdominal plain radiograph in standing posture showed that the multiple air-fluid levels became much few but the “whirlpool” was still in the right iliac fossa (Fig. 1B). Then, the treatment regime was replaced with intravenous immunoglobulin (IVIG) 25 g a day for 5 days and oral prednisone 60 mg a day was taken constantly for 1 month and reduced gradually. Four weeks after the beginning of treatment, her motor and sensory dysfunction improved significantly. She could walk independently for a distance of about 5 m, and the numbness disappeared on the upper limbs and left mildly on the lower limbs. The band feeling on the chest and abdomen almost disappeared. There was no abdominal pain or distention and no obvious constipation. But the patient refused to accept re-examination of abdominal radiograph. Re-examination of CSF showed cell number, protein, and immunoglobulins improved significantly (Table 1).

**Table 1:**
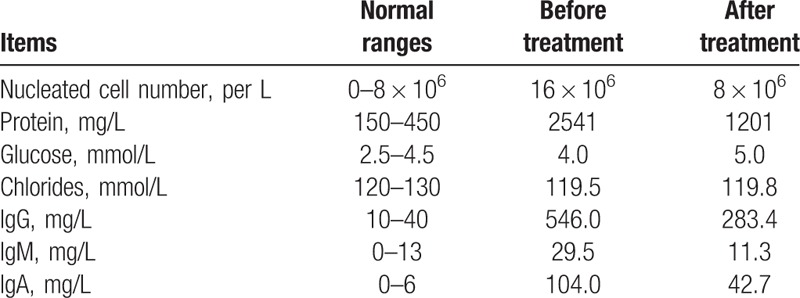
Cerebrospinal fluid examinations before and after treatment for 4 weeks.

This study was approved by the ethics committee of the General Hospital of Wanbei Coal and Electrical Group, and a written informed consent was obtained from the patient for the publication of the case report.

## 3 Discussion

Our patient showed chronic and progressive motor weakness and sensory disturbance with weakened tendon reflex, CSF albuminocytologic dissociation, delayed MNCV, and significant improvement after steroid and IVIG treatment. These findings indicated a CIDP.^[6]^ The patient also had a band feeling on the chest and abdomen which usually implicates spinal cord lesion, but the normal cervical and thoracic MRI and detected peripheral neurologic signs excluded the involvement of spinal cord though the spinal cord, especially the thoracic spinal cord, demyelination occasionally occurs in CIDP.^[7]^ Thus, the band feeling on the chest and abdomen might be due to the sensory disturbance of the inflammatory neuropathy of thoracic and lumbar spinal nerves. Before the onset of somato-symptoms of CIDP and during its course, there was an antecedent and persistent abdominal symptoms presenting as chronic abdominal pain, distension, and constipation, which in combination with the multiple small bowel air-fluid levels and a “whirlpool” image in the abdominal radiograph suggested an incomplete intestinal obstruction.^[8]^ The relapsing of intestinal obstruction after short duration of remission induced by gastrointestinal decompression and almost a complete relief after steroid and IVIG treatment indicated that the incomplete intestinal obstruction was an autonomic dysfunction of CIDP. There are many other causes including diabetes, immune disease, paraneoplastic syndrome, amyloidosis, hereditary, and infectious and toxic diseases that can induce chronic neuropathy with autonomic dysfunction.^[9]^ But the history, blood biochemical results, and the CSF albuminocytologic dissociation in this patient had excluded many causes. Amyloid polyneuropathy may have features mimic to CIDP with autonomic dysfunction including gastrointestinal symptoms of abdominal pain and distention, and the albuminocytologic dissociation in CSF usually can be detected in patients with amyloid polyneuropathy.^[10,11]^ Hence, amyloid polyneuropathy should be differentiated with CIDP in our patient. But the well response to steroid and IVIG treatment and no other organ damage in our patient indicated that the presentations was not caused by amyloidosis though neuropathology was not conducted, as amyloid polyneuropathy has no response to steroid or IVIG treatment.^[10,11]^ Thus, the presentations of this patient should be diagnosed as CIDP with autonomic symptom of incomplete intestinal obstruction.

Autonomic involvement in CIDP is not uncommon, with a prevalence of 21% to 86%, but mostly transient and mild or subclinical.^[2,12–14]^ To our knowledge, this is the first report that a severe autonomic symptom presented as an initial and long-lasting intestinal obstruction in CIDP, and the intestinal obstruction was an isolated clinical presentation of CIDP autonomic polyneuropathy, which usually has grouped symptoms such as cardiovagal, sudomotor, vasomotor, genitourinary, and gastrointestinal events.^[2]^ The mechanism of autonomic dysfunction in CIDP was still unclear. It is speculated that sympathetic and parasympathetic neurons were damaged by the circulating antibodies in the blood, resulting in abnormal synthesis of norepinephrine, acetylcholine, and regulatory peptide that lead to series of autonomic dysfunction, such as cardiac arrhythmia, sweating, gastrointestinal dysfunction.^[15]^

Glucocorticoid is the first choice for CIDP treatment and CIDP generally responds well to IVIG.^[6]^ Some studies had shown that treatment with combination of glucocorticoid and IVIG could improve efficacy and reduce adverse reactions, such as infection caused by high dose of glucocorticoid in CIDP.^[16]^ For our patient, the combined treatment with glucocorticoid and IVIG not only gave rise to a significant improvement of clinical symptoms including motor and sensory disturbances and intestinal obstruction but also improvement of CSF indicators.

## 4 Conclusion

We reported a very unusual CIDP case of which the autonomic symptoms of incomplete intestinal obstruction developed initially and the somato-symptoms developed later, and the incomplete intestinal obstruction persisted long time until the treatment with high dose of glucocorticoid. The diagnosis of CIDP was delayed until the typical CIDP somato-symptoms fully developed. This report highlights the need for diagnostic vigilance in cases of incomplete intestinal obstruction of unknown cause. We recommend CSF and electromyography examination in view of rare but possibility of CIDP.

## Acknowledgments

This study is supported by the Natural Science Grants to Yu Wang (Grant No. 81671290) from the National Natural Science Foundation of China.

## Author contributions

**Resources:** Long Wang.

**Supervision:** Yu Wang.

**Validation:** Long Wang, Xiao-Zheng Yuan, Xue-Min Zhao, Fu-Yu Wang.

**Writing – original draft:** Long Wang.

**Writing – review & editing:** Yu Wang.
